# Plastic Syringes Instead of Heparin-added Dedicated Syringes for Blood Gas Analysis: A Prospective Observational Study

**DOI:** 10.31662/jmaj.2022-0186

**Published:** 2023-03-13

**Authors:** Jumpei Ushikai, Akihiro Tokushige, Hirokazu Shimono, Keisuke Kusumoto, Yoshiyuki Ikeda, Mitsuru Ohishi

**Affiliations:** 1 Department of Internal Medicine, Kagoshima Prefectural Kanoya Medical Center, Kanoya, Japan; 2 Department of Cardiovascular Medicine and Hypertension, Graduate School of Medical and Dental Sciences, Kagoshima University, Kagoshima, Japan; 3 Department of Prevention and Analysis of Cardiovascular Diseases, Graduate School of Medical and Dental Sciences, Kagoshima University, Kagoshima, Japan

**Keywords:** Blood gas analysis, Medical economy, Intensive care unit

## Abstract

**Introduction:**

Blood gas analysis is an important test for making quick and important clinical decisions, and it is recommended that a dedicated syringe that contains heparin be used to measure blood gas. We hypothesized that a plastic syringe could be used as a less-expensive substitute for a dedicated syringe, given that the test is performed immediately after collection.

**Methods:**

This single-center, prospective, observational study involved patients admitted to the Kanoya Medical Center (Kagoshima, Japan) between July 2020 and March 2021, who were requiring blood gas analysis using a dedicated syringe under arterial line (A-line) monitoring. There were no exclusion criteria. Two samples were collected from each patient using a dedicated syringe, and one sample was collected using a plastic syringe. To determine clinical substitutability, Bland-Altman analysis was performed.

**Results:**

A total of 60 samples from 20 consecutive patients were collected and assayed. The mean patient age was 72 years, and 75% patients were men. The 95% limit of agreement for pH, PCO_2_, PO_2_, Na, K, Ca, and SO_2_ were similar for both dedicated and plastic syringes. HCO_3_ and BE were significantly higher in the samples taken with plastic syringes, whereas Hb and Ht could not be measured accurately with any syringe.

**Conclusions:**

The use of plastic syringes in place of dedicated syringes is generally considered acceptable for most items considering that measurement is performed within 3 min of collection, and the cost of medical materials may be reduced. Regardless of the type of syringe, caution should be exercised in interpreting the results of measuring Hb and Ht using a blood gas analyzer.

## Introduction

In the clinical practice of emergency medicine, blood gas measurement contributes to the diagnosis and treatment of cardiovascular, respiratory, and metabolic disorders, in particular.

Blood gas analysis is used not only to evaluate respiratory failure and acid-base balance abnormalities in patients but also to measure electrolytes, hemoglobin, and metabolic parameters in recent years ^(1)^.

It is recommended that specimens for blood gas analysis be collected using a dedicated syringe that contain heparin and be measured as soon as possible ^(2)^. A dedicated syringe should be used to improve the accuracy of the test and to prevent instrument failure by preventing sample coagulation and air inclusion. However, dedicated syringes that contain heparin are relatively very expensive compared to nonheparinized plastic syringes. Therefore, we hypothesized that under conditions where coagulation does not occur (e.g., when measurements are taken within minutes of specimen collection), a nonheparinized plastic syringe could be less-expensive substitute for a dedicated syringe, if it were possible to perform accurate blood gas analysis. Several reports have examined the material of the syringes and the time required for measurement ^(3), (4)^. Nevertheless, the presence or absence of anticoagulants in the syringes has not been previously reported Accordingly, in this study, we investigated the usefulness of blood gas analysis using nonheparinized plastic syringes compared to dedicated syringes.

## Materials and Methods

### Study design

This prospective observational study involved a single institution (Kanoya Medical Center in Kagoshima, Japan), which is a referral type hospital with 186 beds. the clinical ethics committee of the hospital approved the study, which was conducted according to the ethical guidelines for clinical research of the Japanese Ministry of Health, Labour and Welfare and the Declaration of Helsinki. All participants provided written consent.

### Study subjects

This study included 20 consecutive patients admitted to our intensive care unit between July 2020 and March 2021, who were requiring blood gas analysis using a dedicated syringe under A-line monitoring. There were no exclusion criteria. A total of 60 samples, one from each syringe, were collected and assayed from 20 consecutive patients between July 2020 and March 2021.

### Protocol

**Figure 1.**
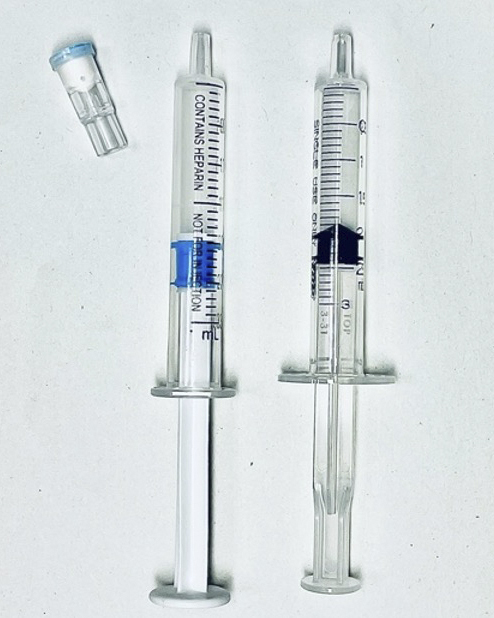
Dedicated syringe (left) and nonheparinized plastic syringe (right).

**Figure 2.**
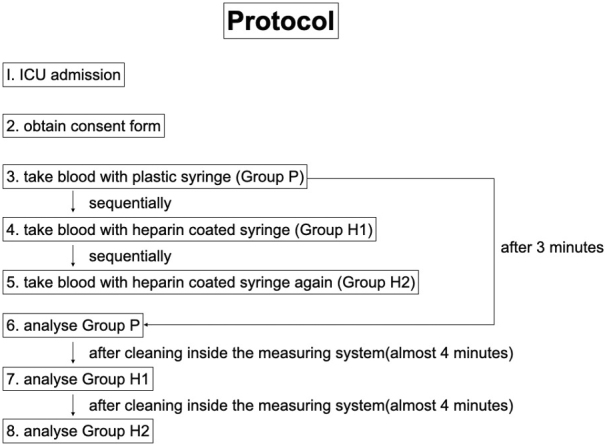
Study protocol.

From the A-line route, one specimen was collected from each patient using a nonheparinized plastic syringe (designated Group P), and two specimens were collected using a dedicated heparinized syringe (designated Groups H1 and H2 in the order in which they were collected). We used TERUMO PrezaPack II^Ⓡ^ as a dedicated syringe (Figure 1). Group P samples were measured within 3 min of collection, and after the analysis was completed and the equipment was cleaned (it takes approximately 4 min from the start of measurement of one sample to the measurement of the next sample), Groups H1 and H2 samples were measured sequentially using the same analyzer (Figure 2). The blood gas analyzer used for the measurements was a GEM^Ⓡ^ Premier 3000 from Instrumentation Laboratory. The items measured were pH, PCO_2_, PO_2_, HCO_3_, Na, K, Ca, Hb, Ht, SO_2_, and BE. We analyzed the results between Groups H1 and H2 and between Groups H1 and P and examined whether plastic syringes could be utilized instead. The frequency of abnormalities in the blood gas analyzers was also observed.

### Clinical data collection

We collected patient background data by medical chart review: smoking was defined as a history of smoking for more than 5 years; hypertension was defined as systolic blood pressure of 140 mmHg or higher or antihypertensive medication; diabetes was defined as a fasting blood glucose of 126 mg/dL or higher and HbA1c of 6.4% or higher or use of medication; anemia was defined as Hb 13 g/dL or less for men and Hb 12 g/dL or less for women; CKD was defined as eGFR 60 mL/min/1.73 m^2^ or less; COPD was categorized as reported or treated; malignancy was defined as with active; heart disease was categorized as ischemic heart disease, congestive heart failure, and pulmonary embolism; lung disease was defined as acute respiratory failure of noncardiac origin; and infectious disease was defined as sepsis, pneumonia.

### Statistical analysis

Based on the difference of the distribution, continuous variables were expressed as mean ± standard deviation (SD) or median and quartile, respectively. Bland-Altman analysis was performed to calculate the 95% limit of agreement (LOA) between Groups H1 and P for each measurement item; if the 95% LOA fell within the predetermined allowable error, it was deemed to be clinically replaceable. Bland-Altman analysis was also performed to compare Groups H1 and H2 for reference. The values of each sample that exceeded 2 SDs of the total were excluded as outliers. The allowable errors were set according to the CLIA performance criteria ^(5)^ for pH, PCO_2_, PO_2_, Na, K, Hb, and Ht. Since there were no CLIA performance criteria for HCO_3_, Ca, SO_2_, and BE, the difference between the upper and lower limits of normal values × 0.4 (which is approximately the same or smaller when applied to other items for which CLIA performance criteria exist) was set as the allowable error for convenience (Table 1).

**Table 1.** Allowable Errors.

	Allowable error	Unit	Decided methods
pH	±0.04		CLIA performance criteria
PCO_2_	±5	mmHg	CLIA performance criteria
PO_2_	±7	mmHg	CLIA performance criteria
HCO_3_	±1.6	mmol/L	{(ULN 26) − (LLN 22)} × 0.4
Na	±4	mmol/L	CLIA performance criteria
K	±0.5	mmol/L	CLIA performance criteria
Ca	±0.08	mmol/L	{(ULN 1.35) − (LLN 1.15)} × 0.4
Hb	±1.12	g/dL	CLIA performance criteria
Ht	±3	%	CLIA performance criteria
SO_2_	±1.6	%	{(ULN 99) − (LLN 95)} × 0.4
BE	±1.6	mmol/L	{(ULN 2) − (LLN −2)} × 0.4

PCO_2_, partial pressure of carbon dioxide; PO_2_, partial pressure of oxygen; HCO_3_, bicarbonate; Na, sodium; K, potassium; Ca, calcium; Hb, hemoglobin; Ht, hematocrit; SO_2_, oxygen saturation; BE, base excess; ULN, upper limit of normal; LLN, lower limit of normal.

JMP 16 pro (SAS, Cary, NC, USA) and R (version 4.0.2; The R Foundation for Statistical Computing, Vienna, Austria) were used for statistical analysis. Statistical significance was assumed at a P-value of <0.05.

### Ethics and dissemination

This study has received ethics approvals from clinical ethics committee of Kanoya Medical Center (No. 2001). All participants provided written consent.

## Results

### Patient characteristics

Table 2 shows the patient characteristics. The mean age of patients was 72 years. In total, 75% patients were men, 70% had hypertension, 60% had diabetes mellitus, 55% had cardiac disease, 25% had pulmonary disease, and 15% had an infectious disease.

**Table 2.** Patient Characteristics.

Variables	
Age, years	72 ± 14.5
Male, N	75%
BMI, kg/m^2^	24 ± 12.4
Smoking, N	65%
SBP, mmHg	122 ± 28.9
HTN, N	70%
DM, N	60%
Anemia, N	40%
Hb, N	12.5 ± 1.95
CKD, N	25%
COPD, N	30%
Malignancy, N	10%
Ventilator, N	20%
Antiplatelet therapy, N	40%
Anticoagulation therapy, N	10%
Plt, ×10^4^/μL	18.8 ± 5.8
PT-INR	1.10 ± 0.11
Cause of admission
Heart disease, N	55%
Lung disease, N	25%
Infectious disease, N	15%
Others, N	5%

N indicates number. The normal distribution continuous values are shown as mean ± SD. BMI, body mass index; SBP, systolic blood pressure; HTN, hypertension; DM, diabetes mellitus; Hb, hemoglobin; CKD, chronic kidney disease; COPD, chronic obstructive pulmonary disease; Plt, platelets.

### Bland-Altman analysis

**Figure 3.**
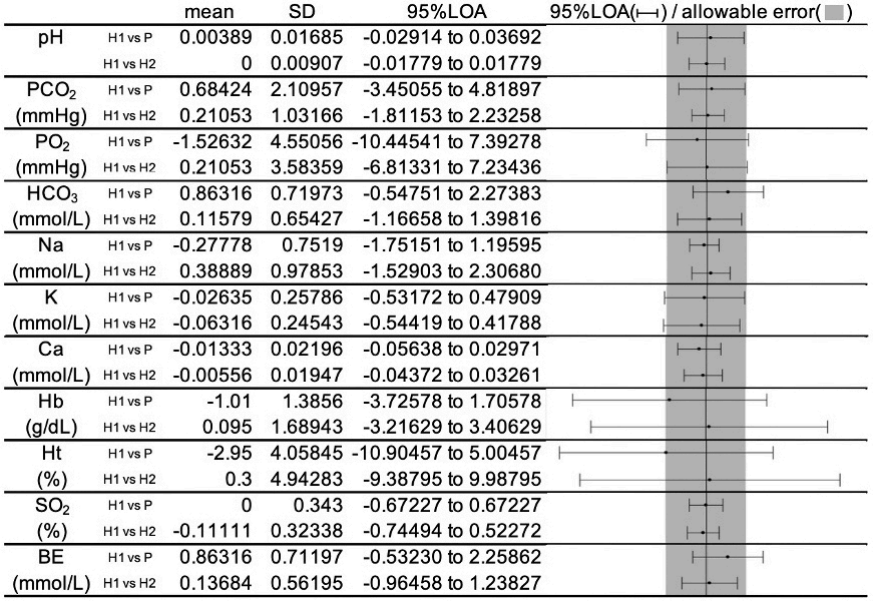
Mean, standard deviation, and 95% LOA of the difference between “Groups H1 and P” and “Groups H1 and H2” for pH, PCO_2_, PO_2_, HCO_3_, Na, K, Ca, Hb, Ht, SO_2_, and BE.

The mean, SD, and 95% LOA of the difference between “Groups H1 and P” and “Groups H1 and H2” for each item (Figure 3), and Supplementary Materials S1-S11 show the Bland-Altman plot.

For pH, PCO_2_, Na, Ca, and SO_2_, the 95% LOA was within the predetermined allowable error for “Groups H1 and P.”

The 95% LOA of PO_2_ and K slightly exceeded the allowable error in “Groups H1 and P” and similar findings were obtained in “Groups H1 and H2.”

For Hb and Ht, the 95% LOA greatly exceeded the allowable error in “Groups H1 and P,” but the same finding was also obtained in “Groups H1 and H2.”

The 95% LOA of HCO_3_ and BE exceeded the allowable error in “Groups H1 and P” but not in “Groups H1 and H2.”

There were no errors or failures of the measuring instruments during the observation period.

## Discussion

### Key findings

The ﬁndings of this study were as follows: (a) The use of plastic syringes in place of dedicated syringes is generally considered acceptable for pH, PCO_2_, PO_2_, Na, K, Ca, and SO_2_ if measurements are performed within 3 min of collection, and (b) the interpretation of Hb and Ht measured using a blood gas analyzer should be interpreted with caution irrespective of the type of syringe.

### Plastic syringes versus dedicated syringes

Knowles et al. investigated whether the gas analysis results changed depending on the material of the syringe, the temperature of the specimen, and the time until measurement ^(3)^. Nevertheless, the purpose of the authors’ enquiry is different from that of the present study in which anticoagulants were used in all samples and electrolytes were not measured. However, in the group that was compared immediately after specimen collection, the measured values did not differ according to the syringe material or temperature. Further reports have verified the temperature and time to measurement, and the results of the gas analysis become less reliable as the time to measurement increases ^(4), (6), (7)^. There are no published reports on blood gas measurements including electrolytes and the presence or absence of anticoagulants. In this respect, the results of this study are the first report of their kind.

In this study, the 95% LOA of pH, PCO_2_, Na, Ca, and SO_2_ between Groups H1 and P was within the allowable error range, suggesting that a plastic syringe can be used as a substitute a dedicated syringe under the present conditions, in which measurement of these items should be started within 3 min of collection. Additionally, the 95% LOA of PO_2_ and K between the two groups of Groups H1 and P was slightly above the allowable error range, although comparable results were observed between Groups H1 and H2. The difference between the two Bland-Altman plots was small, which suggests that the difference between the two groups is clinically acceptable.

In the comparison between Groups H1 and P, HCO_3_ and BE were significantly higher in Group P, and 95% LOA was also skewed upward and exceeded the range of allowable error, suggesting the presence of a fixed error. This finding was not observed between Groups H1 and H2. The cause of this systematic error may be attributed to the effect of lithium heparin, which is added to the samples in Groups H1 and H2, where the pH range is 5.0-8.0, and the samples are expected to be more acidic, resulting in higher values of HCO_3_ and BE in Group P. A similar trend was also observed for pH, although to a lesser extent. Therefore, when measuring HCO_3_ and BE using plastic syringes, care should be taken in interpreting the results.

### Hb and Ht

For Hb and Ht, the 95% LOA was well above the allowable error range in the comparison between Groups H1 and P. Nevertheless, same results were also observed between Groups H1 and H2, suggesting that blood gas analyzers may not be able to measure Hb and Ht with high reliability irrespective of the type of syringe. In fact, there are several reports comparing Hb values of blood gas analysis and peripheral blood tests ^(8), (9)^, although these do not show such a large difference as that observed in this study. A possible mechanism for the inaccurate Hb measurement in this study may be the effect of precipitation of blood cells due to insufficient mixing and agitation. However, we postulate that both plastic and heparin syringes failed to measure Hb and Ht accurately in this study, and this is not a significant concern in the discussion of whether plastic syringes can be substitutes for heparin-added dedicated syringes.

### Clinical implications

This study is the first to report on the usefulness of blood gas analysis using nonheparinized plastic syringes compared to heparin-added dedicated syringes. In our country, plastic syringes are 10-20 times less expensive than dedicated syringes. Therefore, blood gas analysis without the use of dedicated syringes will reduce costs and contribute to medical economy in several clinical situations.

### Study limitations

Some limitations of this study should be noted. First, the specimens in this study were measured with a single model analyzer and a single model syringe, so we are unable to establish the results that would be obtained using other manufacturers’ analyzers and syringes. However, TERUMO PrezaPack II, which was used in this study, has a large share of the Japanese market, and these results are applicable to clinical practice in many facilities.

The second, Bland-Altman analysis ^(10)^, is a popular method for evaluating the equivalence of two tests, but there are no established criteria for determining the equivalence of two tests for each analysis. In most cases, the decision is based on the intended application, a comparison with similar measurement methods that have already been deemed equivalent, or a consensus among experts ^(11)^. In the present study, we compared the Bland-Altman plot of two samples collected with a dedicated syringe as an equivalent measurement method and employed the CLIA performance criteria as an expert consensus. However, there were no CLIA performance criteria for SO_2_, HCO_3_, Ca, and BE. We set the allowable error for these parameters using a formula that is generally equivalent to or more stringent than the CLIA performance criteria, and we postulate that many clinicians will agree on the equivalence of these items.

Finally, because the sample size is relatively small, further verification using a larger sample size is required before the results can be applied to actual clinical practice. In terms of safety, in the present study, no errors or malfunctions were observed in the measuring instruments, and we conclude that the use of plastic syringes is safe, but because the sample size for instrument malfunctions was small, an accurate evaluation may not be possible.

### Conclusions

This study suggests that the use of plastic syringes instead of heparin-added syringes is generally considered acceptable for the majority of specimens as long as the measurement is performed within 3 min of collection.

However, regardless of the type of syringe, caution must be taken while interpreting the results of measuring Hb and Ht using a blood gas analyzer.

Verification with more models, types of syringes, and number of specimens may reduce the cost of medical materials in settings where blood gas analysis is performed frequently, such as emergency rooms, operating rooms, and intensive care units.

## Article Information

### Conflicts of Interest

None

### Acknowledgement

The authors would like to acknowledge the valuable contribution of the clinical research coordinators at Kanoya Medical Center.

### Author Contributions

Manuscript concept and design: Jumpei Ushikai and Akihiro Tokushige; writing: Jumpei Ushikai and Akihiro Tokushige; data collection: Jumpei Ushikai, Hirokazu Shimono, and Keisuke Kusumoto; statistical analyses: Jumpei Ushikai and Akihiro Tokushige; revision and editing: Hirokazu Shimono, Keisuke Kusumoto, Yoshiyuki Ikeda, and Mitsuru Ohishi; acceptance of the ﬁnal version: all authors.

### Approval by Institutional Review Board (IRB)

Clinical ethics committee of Kanoya Medical Center (No. 2001)

## Supplement

Supplementary Materials
