# Supplementary material for: Plastic Syringes Instead of Heparin-added Dedicated Syringes for Blood Gas Analysis: A Prospective Observational Study
Source: JMA J. 2023 Mar 13;6(2):175–81. doi: 10.31662/jmaj.2022-0186 (PMC10169280; doi:10.31662/jmaj.2022-0186)

SUPPLEMENTARY MATERIAL

Figures

Figure S1.

Bland–Altman plot between "Group H1 and Group P" and "Group H1 and Group H2" for pH (—means 95% LOA, and ---- means allowable error, hereafter the same shall apply)

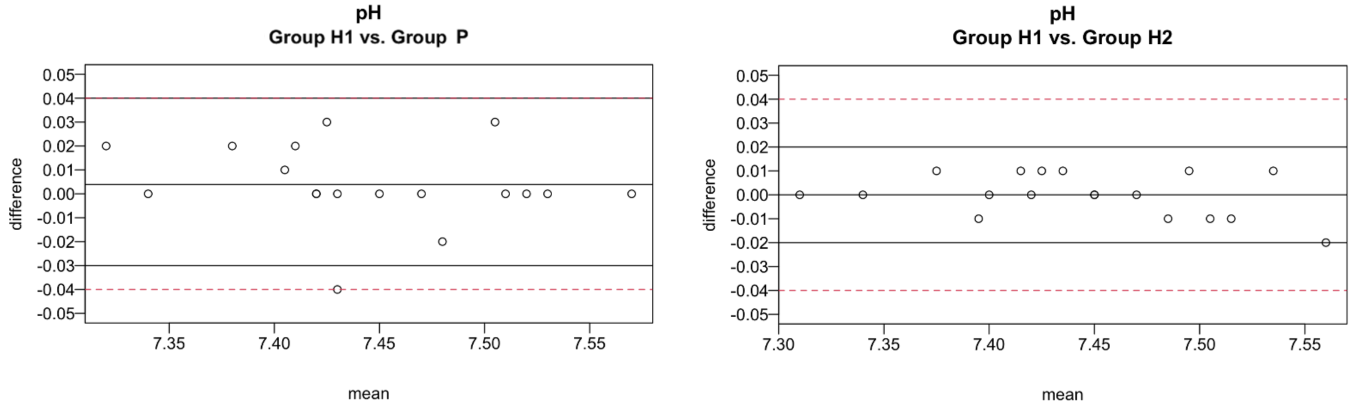

Figure S2.

Bland–Altman plot between "Group H1 and Group P" and "Group H1 and Group H2" for PCO_2

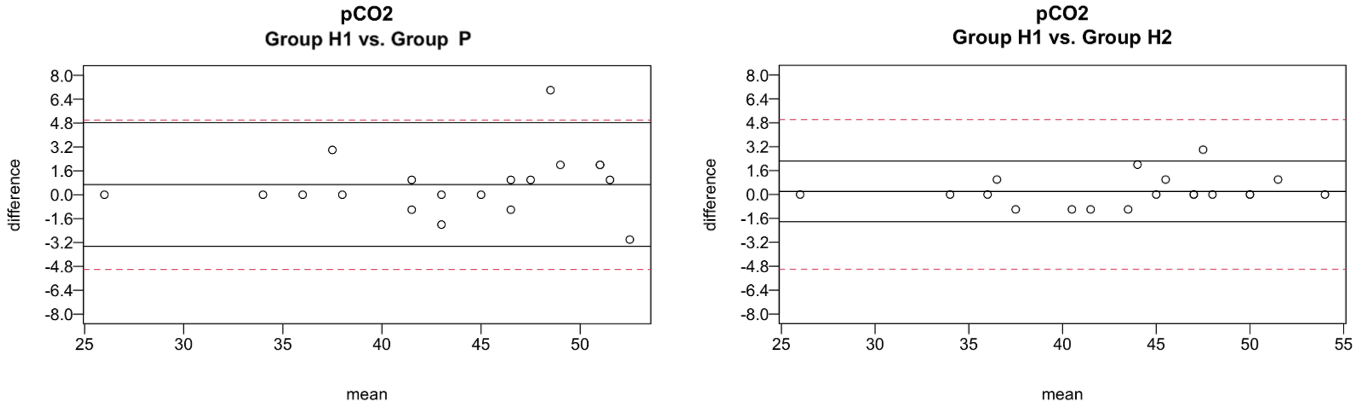

Figure S3

Bland–Altman plot between “Group H1 and Group P” and “Group H1 and Group H2” for PO_2

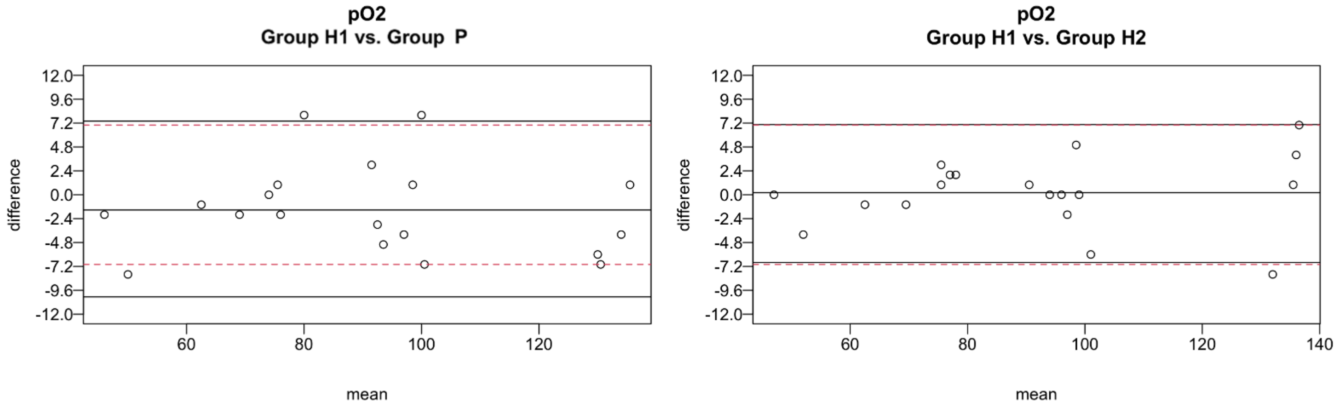

Figure S4

Bland–Altman plot between “Group H1 and Group P” and “Group H1 and Group H2” for HCO_3

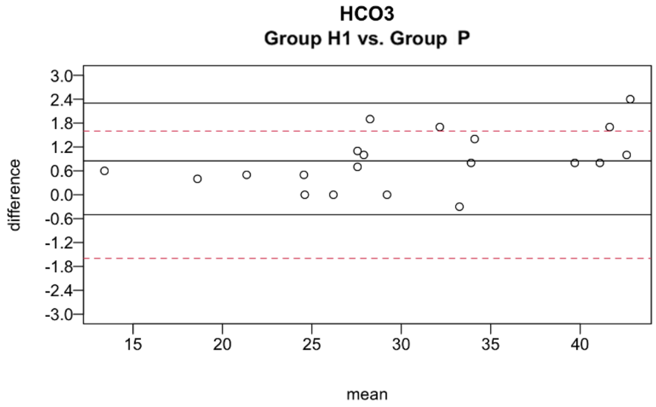

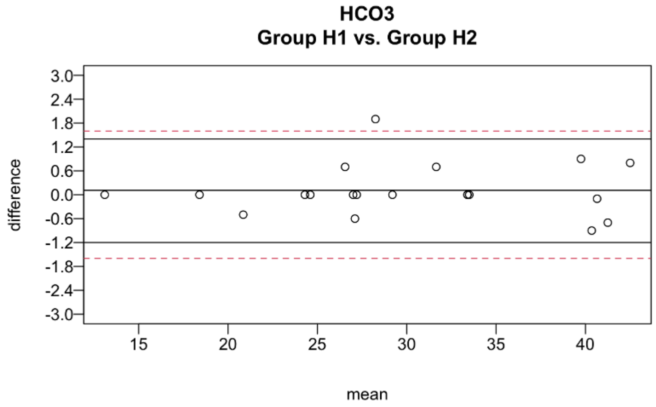

Figure S5

Bland–Altman plot between “Group H1 and Group P” and “Group H1 and Group H2” for Na

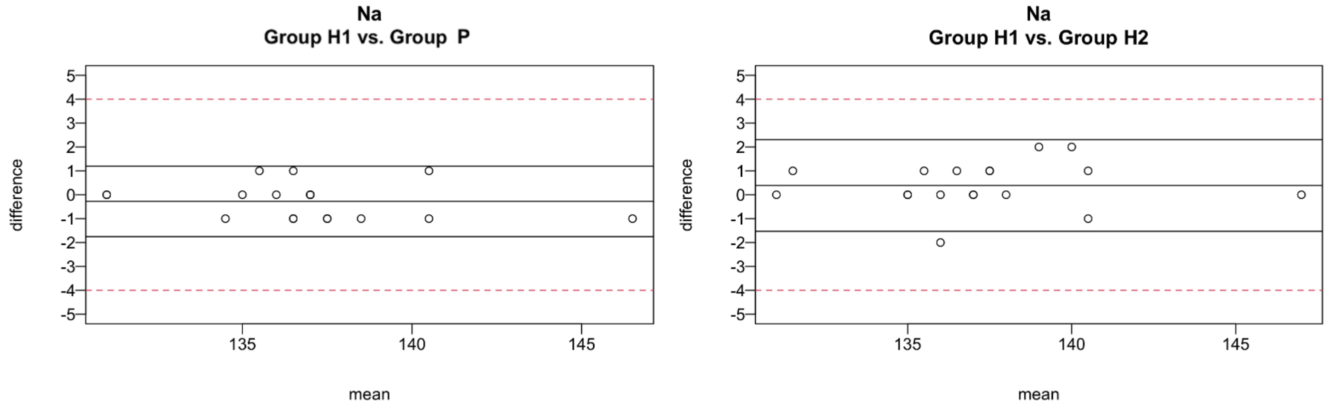

Figure S6

Bland–Altman plot between “Group H1 and Group P” and “Group H1 and Group H2” for K

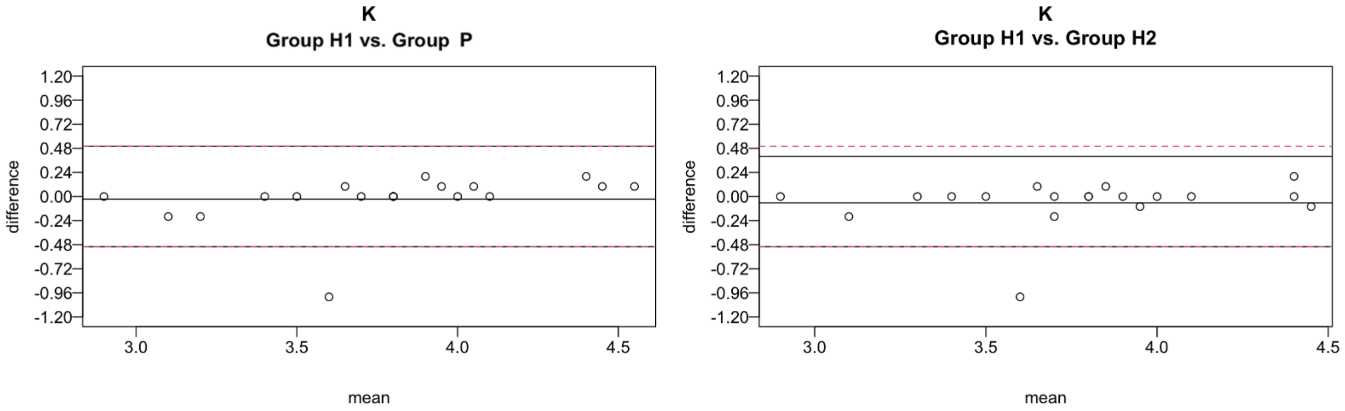

Figure S7

Bland–Altman plot between "Group H1 and Group P" and "Group H1 and Group H2" for Ca

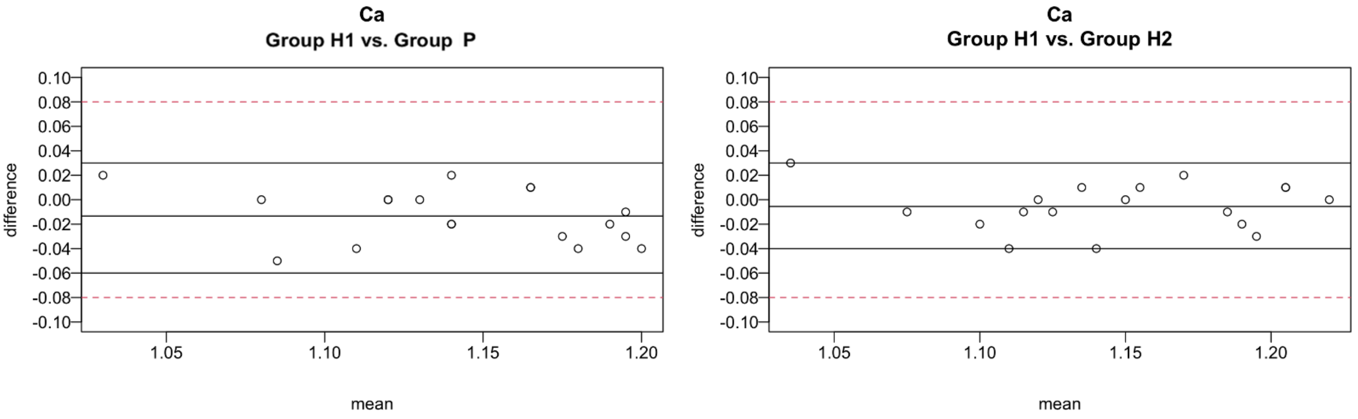

Figure S8

Bland–Altman plot between “Group H1 and Group P” and “Group H1 and Group H2” for Hb

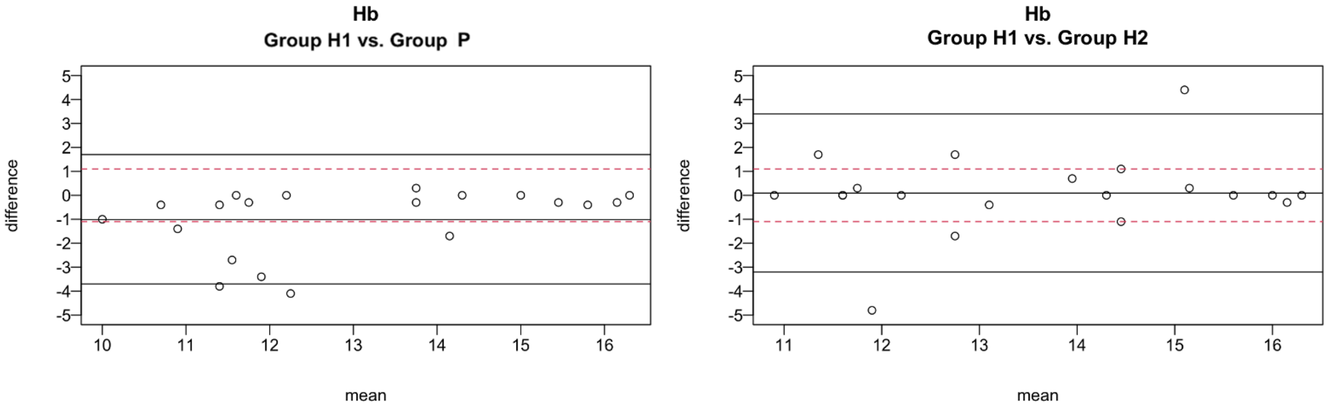

Figure S9

Bland–Altman plot between "Group H1 and Group P" and "Group H1 and Group H2" for Ht

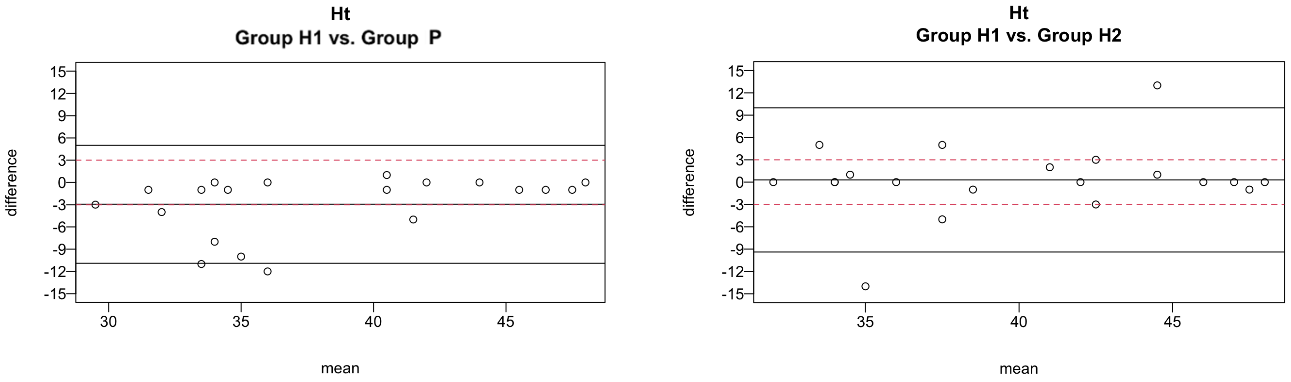

Figure S10

Bland–Altman plot between "Group H1 and Group P" and "Group H1 and Group H2" for SO_2

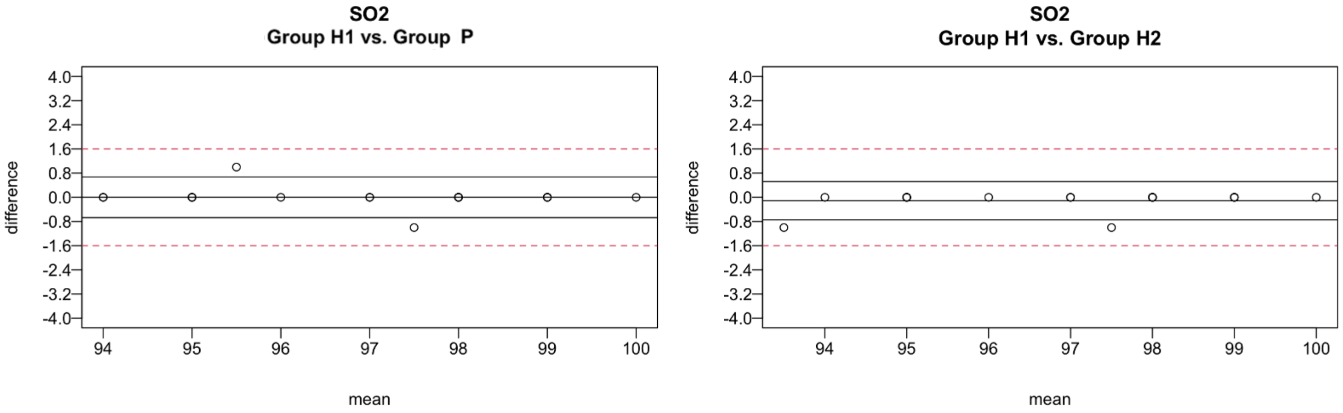

Figure S11

Bland–Altman plot between “Group H1 and Group P” and “Group H1 and Group H2” for BE

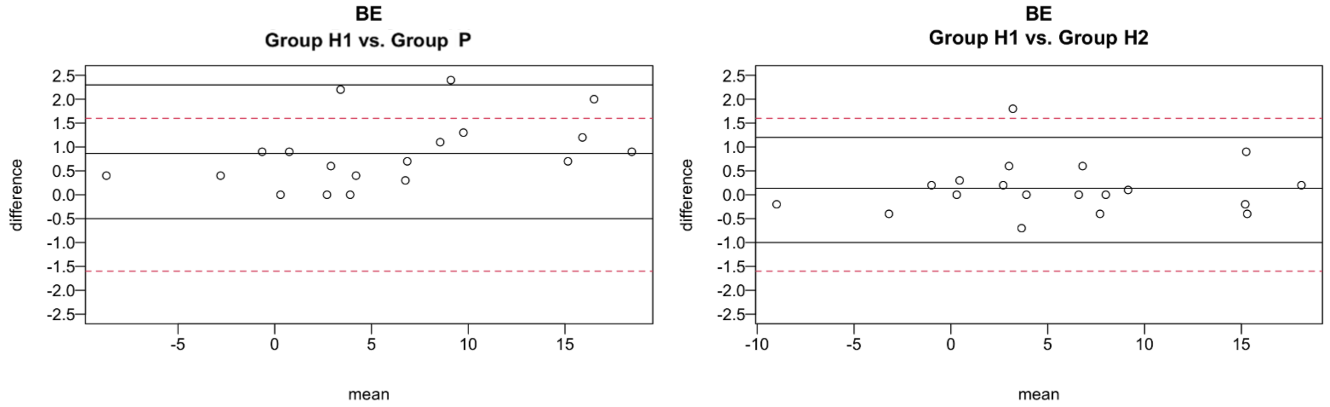

Supplement: Supplementary Materials [file 2433-3298-6-2-0175-s001.pdf]
